# Supplementary material for: Comparative Evaluation of Large Language and Multimodal Models in Detecting Spinal Stabilization Systems on X-Ray Images
Source: J Clin Med. 2025 May 8;14(10):3282. doi: 10.3390/jcm14103282 (PMC12112668; doi:10.3390/jcm14103282)
Supplement: Supplementary file 1 [file jcm-14-03282-s001.zip › jcm-3550108-supplementary.pdf]

Supplementary table S1: Detailed detection levels and inter-rater agreement for GPT-4o in detecting spinal stabilization systems (SSS) in advanced scoliosis (≥40°) using posturographic radiographic images

SSS Type	Classification Task	n_{obs}	Proper Response	Rater 1, n (%)	Rater 2, n (%)	Rater 3, n (%)	pa	pe	AC1	SE	95% CI	p-value
**No Stabilization**	Presence	93	None/not applicable	92 (98.92%)	89 (95.70%)	89 (95.70%)	0.97	0.06	0.97	0.02	0.94, 1.00	< 0.001
	Type	93	None/not applicable	92 (98.92%)	89 (95.70%)	89 (95.70%)	0.97	0.06	0.97	0.02	0.94, 1.00	< 0.001
	Kind	93	None/not applicable	92 (98.92%)	89 (95.70%)	89 (95.70%)	0.97	0.06	0.97	0.02	0.94, 1.00	< 0.001
**PSF**	Presence	97	Yes	97 (100%)	97 (100%)	97 (100%)	1.00	1.00	1.00	-	1.00, 1.00	-
	Type	97	Non-growing	95 (97.94%)	96 (98.97%)	95 (97.94%)	0.99	0.03	0.99	0.01	0.97, 1.00	< 0.001
	Kind	97	PSF	83 (85.57%)	81 (83.51%)	80 (82.47%)	0.90	0.27	0.87	0.04	0.80, 0.94	< 0.001
**MCGR**	Presence	80	Yes	80 (100%)	80 (100%)	80 (100%)	1.00	1.00	1.00	-	1.00, 1.00	-
	Type	80	Growing	71 (88.75%)	65 (81.25%)	33 (41.25%)	0.61	0.42	0.32	0.08	0.16, 0.49	< 0.001
	Kind	80	MCGR	14 (17.50%)	29 (36.25%)	1 (1.25%)	0.65	0.29	0.50	0.07	0.36, 0.64	< 0.001

Notes: n_{obs}: Number of observations (X-ray images). Proper Response: Expected correct response for each classification task. Rater 1, 2, 3: Percentage of correct responses by each rater for GPT-4o.pa : Observed agreement; pe : Expected agreement by chance; AC1: Gwet's Agreement Coefficient 1; SE: Standard error; 95% CI: Confidence interval for AC1; p-value: Statistical significance of AC1. BiomedCLIP's performance is not rater-based (perfect agreement, AC1 = 1.00) and is reported in the main manuscript (e.g., sensitivity: 99.6% for No Stabilization Presence, 5.1% for PSF Presence, 6.6% for MCGR Presence). Refer to Table 2 for comparative sensitivity values.Data are derived from repeated analyses of 270 posturographic X-ray images (93 No Stabilization, 97 PSF, 80 MCGR) conducted between January 1 and January 30, 2025.
